# High Transmission Efficiency Hybrid Metal-Dielectric Metasurfaces for Mid-Infrared Spectroscopy

**DOI:** 10.3390/nano15181456

**Published:** 2025-09-22

**Authors:** Amr Soliman, Calum Williams, Timothy D. Wilkinson

**Affiliations:** 1 Electrical Engineering Division, Department of Engineering, University of Cambridge, 9 JJ Thomson Avenue, Cambridge CB3 0FA, UK; tdw13@cam.ac.uk; 2 Department of Physics, University of Exeter, Stocker Rd, Exeter EX4 4QL, UK; c.williams15@exeter.ac.uk

**Keywords:** hybrid metal-dielectric metasurfaces, all-dielectric metasurfaces, plasmonics, MIR spectroscopy

## Abstract

Mid-infrared (MIR) spectroscopy enables non-invasive identification of chemical species by probing absorption spectra associated with molecular vibrational modes, where spectral filters play a central role. Conventional plasmonic metasurfaces have been explored for MIR filtering in reflection and transmission modes but typically suffer from broad spectral profiles and low efficiencies. All-dielectric metasurfaces, although characterized by low intrinsic losses, are largely limited to reflection mode operation. To overcome these limitations, we propose a hybrid metal-dielectric metasurface that combines the advantages of both platforms while simplifying fabrication compared to conventional Fabry–Pérot filters. The proposed filter consists of silicon (Si) crosses atop gold (Au) square patches and demonstrates a transmission efficiency of 87% at the operating wavelength of 4.28 µm, with a full width half maximum (FWHM) as narrow as 43 nm and a quality factor of approximately 99.5 at λ = 4.28 μm. Numerical simulations attribute this performance to hybridization of Mie lattice resonances in both the gold patches and silicon crosses. By providing narrowband, high-transmission filtering in the MIR, the hybrid metasurface offers a compact and versatile platform for selective gas detection and imaging. This work establishes hybrid metal–dielectric metasurfaces as a promising direction for next-generation MIR spectroscopy.

## 1. Introduction

Mid-infrared (MIR) spectral region—typically defined as spanning approximately 2–20 µm [1]—is a powerful technique for probing molecular absorption features that originate from the fundamental vibrational modes of chemical bonds. Gas-sensing applications, such as the detection of CO, CO_2_, NO, NH_3_, and CH_4_, rely on characteristic MIR absorption bands, and spectroscopic analysis enables their identification with high selectivity [2,3]. MIR spectroscopy is also widely used in chemical analysis as it can be used for the identification of molecular structures that have characteristic MIR absorption ‘fingerprints’ (i.e., unique absorption spectra) [4]. Moreover, many biochemical building blocks including DNA, lipids, and proteins can be identified using MIR spectroscopy [4,5]. To achieve cost-effective detection of specific spectral signatures, rather than full continuous spectral capture, compact MIR spectroscopy designs typically employ Fabry–Perot (FP) filters [6,7,8,9] or plasmonic/all-dielectric metasurfaces [10,11,12,13,14].

Fabry–Pérot bandpass filters consist of two distributed Bragg reflectors separated by a central cavity. The distributed Bragg reflectors provide a reflection stop-band while the central cavity’s optical thickness sets the center wavelength [8]. Despite having high transmission efficiency with high quality factors, Fabry–Pérot filters need multiple microfabrication (thin film physical vapor deposition) steps to deposit different layers which increases the fabrication time, decreases the device compactness, and makes it challenging to construct FP-based multispectral filters on the same chip/spatial area. Moreover, due to the lack of refractive index contrast between layers, Fabry–Pérot filters experience a small stop-band leading to cross-talk between spectral channels, which diminishes the filter performance [7].

Plasmonic metasurfaces have been widely used in visible and MIR spectroscopy [10,11,12,13,14]. Typically, the resonance wavelength of the plasmonic metasurface is adjusted to overlap with the characteristic absorption spectra of the molecular structure or the biochemical building block to be identified. This overlap between the plasmonic metasurface resonance and the characteristic absorption fingerprint changes the strength of the plasmonic resonance. The molecular structure or the gas can be identified by comparing the metasurface spectra before and after the change in the response [4]. However, plasmonic metasurfaces experience different problems that affect their performance in MIR spectroscopy. For instance, despite the fact that plasmonic metasurfaces exhibit strong local electric field enhancement compared to the excitation field, they exhibit high Joule losses due to the absorption of the plasmonic metals, resulting in a low Q-factor response with low efficiency. While such broad, low-Q resonances can be acceptable in many visible and near-infrared applications (e.g., imaging, color filtering, and SERS), they are particularly detrimental in MIR spectroscopy, where resolving sharp molecular absorption lines (e.g., CO_2_ at 4.26 µm, CO at 4.67 µm) requires high spectral selectivity. In this case, broad plasmonic resonances lead to poor discrimination of absorption features and reduced sensing accuracy. In addition, plasmonic metasurfaces experience cross-talk due to the excitation of additional resonances in the same operating wavelength range [10].

To overcome these limitations, all-dielectric metasurfaces have emerged as promising alternatives because of their reduced intrinsic losses and ability to support both electric and magnetic resonances, offering greater design flexibility [15]. By mitigating the efficiency challenges of plasmonic designs, dielectric metasurfaces have recently been employed for MIR detection [4,5]. For instance, a dielectric metasurface array featuring ultra-sharp resonances tailored to distinct wavelengths was used to detect molecular absorption fingerprints across multiple spectral positions, producing a barcode-like absorption pattern [4]. Similarly, another high-Q dielectric metasurface platform demonstrated sensitive molecular identification, though it required additional readout instrumentation and complex image processing [5]. In both cases, the observed high-Q reflection resonances originated from bound states in the continuum (BICs) [4,5,16]. Nevertheless, prior studies have been restricted to reflection-mode operation, since all-dielectric metasurfaces typically produce only transmission dips [17]. This highlights the need for a MIR spectroscopy platform that operates in transmission mode while maintaining high efficiency and spectral selectivity.

Hybrid metal–dielectric metasurfaces have recently attracted significant research interest by combining the advantages of plasmonic and all-dielectric designs while mitigating their respective drawbacks [18,19,20,21]. Hybrid designs aim to mitigate high ohmic absorption (plasmonic) losses and low field enhancement (all-dielectric), which result in higher transmission efficiencies and more controllable transmission responses. Moreover, they are characterized by ultra-narrow bandwidths (high Q-factors [22]) and low cross-talk [15] making them attractive for a wide range of applications.

Hybrid metal-dielectric metasurfaces have been used in transmission mode for different applications in the visible and MIR wavelength ranges operating in transmission mode. For instance, transmission structural colors with an efficiency of up to 70% were generated using hybrid metal-dielectric metasurfaces [17]. The colors were produced through the interaction of aluminum Wood’s anomaly with silicon nitride Mie resonances. Moreover, hybrid metal-dielectric metasurfaces have been used for near-infrared refractive index sensing [18]. However, the reported hybrid metasurface has a low Q-factor. Also, it is exposed to cross-talk due to the excitation of multiple resonant peaks in the same operating wavelength range, reducing its efficiency in filtering and sensing applications. Another hybrid metal-dielectric metasurface working in the transmission mode is used to steer light into a preferential direction with high directionality [19]. However, the spectrum of the reported hybrid metasurfaces does not have any sharp peaks and cannot be used for MIR spectroscopy which requires one or two sharp peaks in the spectrum. In this context, our work is motivated by the need to overcome these shortcomings by introducing a transmission-mode hybrid metasurface bandpass filter that achieves simultaneously high transmission efficiency (87%), a high Q-factor (~99.5), and a narrow FWHM (43 nm). The choice of transmission mode is particularly relevant for practical MIR applications, as it enables straightforward integration into compact optical systems where the transmitted light directly reaches the detector without the need for additional reflective optics. This not only reduces system complexity but also aligns with real-world MIR systems such as multispectral imaging setups [23], compact MIR spectrometers [24], and MIR cameras [25]. By addressing the limitations of prior metasurfaces and tailoring the design to transmission-based operation, our approach provides a viable pathway toward compact, efficient, and high-performance MIR spectroscopy platform.

Recent progress in metasurfaces has demonstrated remarkable advances in optical sensing across ultraviolet to near-infrared regimes. All-silicon metasurfaces have achieved high-Q perfect absorption with ultra-narrow linewidths of 4.6 nm and exceptionally high figures of merit [26]. Rhodium-based ultraviolet nanocavities have shown plasmon-induced reflection with 15 nm linewidths, polarization-tunable field enhancement, and high SERS sensitivity [27]. Thermo-optic metasurfaces have been proposed for multispectral absorption from UV to NIR with dynamic temperature tuning [28], and tunable dual-band absorbers in the ultraviolet–visible (UV–vis) spectrum have also been reported using three-dimensional metamaterials [29]. Beyond these optical regimes, related concepts have long been explored in other parts of the spectrum—for example, microwave and far-infrared frequency-selective surfaces (FSS) [30,31], near-infrared plasmonic C-shaped nano-engravings [32] and polarization-sensitive plasmonic nano-C-apertures together with related near-infrared plasmonic architectures [33,34]. These approaches illustrate the continuity of spectral filtering concepts across wavelengths. In contrast, the present work demonstrates a transmission-mode bandpass filter in the mid-infrared range, optimized for molecular gas sensing.

This work presents a high-transmission hybrid metal–dielectric metasurface bandpass filter operating in the MIR spectrum, achieving a Q-factor of approximately 99.5 at λ = 4.28 µm with a calculated FWHM of 43 nm, thereby surpassing the performance of tunable mid-IR Fabry–Pérot bandpass filters (Q ≈ 70–90; FWHM = 50–65 nm) [6]. The coupling of the Au electric dipole (ED) with the ED and magnetic dipole (MD) resonances of Si induces multiple multipolar responses—including ED, MD, electric quadrupole (EQ), and magnetic quadrupole (MQ), leading to high transmission efficiency and an enhanced Q-factor. The proposed metasurface comprises subwavelength arrays of silicon crosses combined with gold patches, which exhibit a high transmission efficiency (87%), narrow bandwidth (43 nm), minimal spectral cross-talk, and a CWL of λ = 4.28 μm. The CWL matches the corresponding MIR absorption band of CO_2_ [35] for use in gas detection applications. However, we have shown through numerical simulations that by adjusting its geometric dimensions, the metasurface enables spectral tuning across the MIR band to align with the characteristic absorption features of various gases. Hence, the presented hybrid metasurface can be easily adapted for MIR spectroscopy.

## 2. Design and Simulations

### 2.1. Design

The first step in our design was to choose the correct materials for the system. Au is widely used as a plasmonic metal due to its relatively low losses in the MIR and stability against oxidation in ambient air, unlike other metals such as Al and Ag [36].

For the dielectric material, the optical confinement of the metasurface primarily depends on the refractive index contrast with its surrounding medium. As most metasurfaces operate in air (*n* = 1), increasing the refractive index of the structural material enhances the refractive index contrast. Accordingly, materials with high refractive indices help suppress radiation leakage and improve field confinement, even at subwavelength scales [37,38]. In this work, Si was chosen because it combines a high refractive index with low absorption in the MIR region [5]. Moreover, Si is widely used in semiconductor technologies and is compatible with CMOS processing [18]. The complex dispersive refractive indices for Au and Si were taken from the Palik material model available in the Lumerical FDTD built-in library to ensure accurate wavelength-dependent dispersion. The substrate material was chosen to be CaF_2_, attributed to its low refractive index (1.4) and negligible losses in the MIR [39]. The CaF_2_ substrate was modeled with a thickness of 1.1 mm, which is consistent with commercially available substrates.

**Figure 1:**
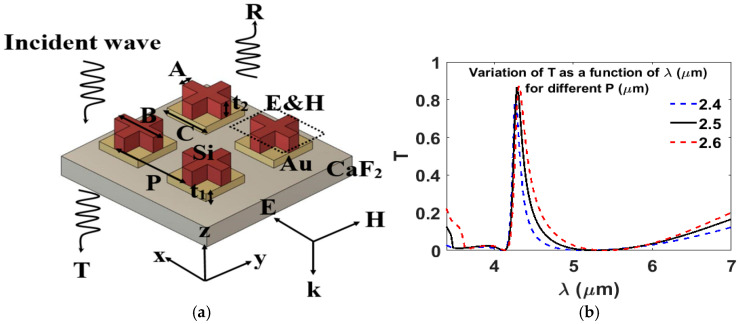

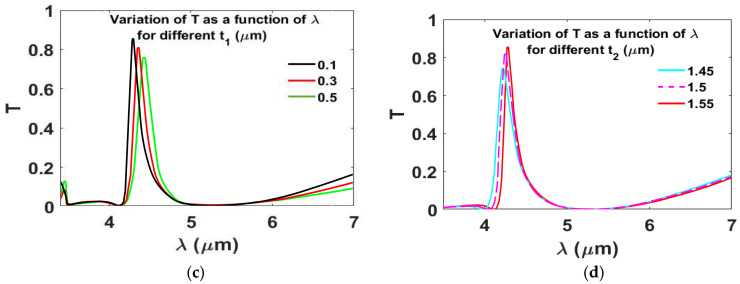
(**a**) Schematic of the proposed filter showing the structural parameters: *P* is the period, *t*_1_ is the Au thickness, *t*_2_ is the Si thickness, *A* and *B* are the width and length of the cross, respectively, and *C* is the length of the Au patch. (**b**–**d**) Simulated transmission spectra illustrating the effect of varying (**b**) the periodicity *P* (**c**) the thickness *t*_1_, and (**d**) the thickness *t*_2_ on the transmission efficiency T. In each case, only one parameter was varied while the others were kept constant. The final optimized parameters are summarized as follows: at *P* = 2.5 μm, *A* = 0.3 μm, *B* = 2.2 μm, *C* = 2.2 μm, *t*_1_ = 0.1 μm, and *t*_2_ = 1.55 μm.

The next step is to choose the design of the metasurface. We chose the Si cross as the response can be controlled via three different geometrical parameters adding an additional degree of freedom compared to nanodisks, which is desirable for optimizing the spectral response. While the Au patch is chosen as it attains the optimal results based on an exploration of the parameter space giving the highest transmission response and a high Q-factor at the operating wavelength of 4.28 μm. A schematic of the proposed hybrid metasurface, along with its geometrical parameters, is shown in Figure 1. Each unit cell of the filter is composed of an Au (plasmonic) patch and Si (dielectric) cross on a CaF_2_ substrate.

### 2.2. Simulations

Electromagnetic simulations were performed using Lumerical (Ansys) FDTD Solutions to analyze and optimize the spectral response of the MIR filter [40]. The goal aimed at achieving a resonant peak with the highest possible transmission efficiency and a narrow FWHM at λ = 4.28 μm. Hence, we simulated the (3D) optical transmission response of varying filter geometries to yield the optimal design. It should be noted that all transmission, reflection, and absorption spectra are normalized to the incident power, and therefore the corresponding y-axes are dimensionless. Furthermore, we calculated the E-and-H field profiles at the resonant wavelength to provide insight into the phenomena underpinning the response. Wideband linearly polarized plane waves were normally incident on the filter geometry. Periodic boundary conditions were applied in the x and y directions in order to emulate an infinite array while perfectly matched layers (PMLs) were used in the z direction to minimize reflections. A mesh size of 5 nm was utilized at the metasurface unit layer boundaries and cross corners for increased accuracy. The complex dispersive refractive indices for Au (Palik) and Si (Palik) were obtained using multi-coefficient models supported by Lumerical FDTD, whilst the non-dispersive refractive index of CaF_2_ substrate was assumed to be 1.4 with no losses in the MIR range [39].

The multipole expansion and scattering cross section (SCS) were simulated using the open-source MATLAB code (MathWorks, R2023a) (*MENP)* [39]. *MENP* calculates the ED, MD, EQ, and MQ contributions and then evaluates the SCS. The electric fields within the metasurface were obtained from Lumerical FDTD and exported in *.mat* format, along with the refractive index *n* (*x*, *y*, *z*, *f*) and one-dimensional axis arrays (*x*, *y*, *z*, *f*). These fields were then imported into *MENP*, which first converts the electric field distributions into current density. Based on this current density, *MENP* computes the four multipole modes (ED, MD, EQ, MQ) and subsequently the SCS. The FWHM was obtained numerically from the simulated transmission spectrum by identifying the wavelengths at which the transmission drops to 50% of its maximum on either side of the resonance peak and taking their difference. The Q-factor was then calculated using Q = λ_0_/FWHM, where λ_0_ is the peak transmission wavelength.

## 3. Results and Discussions

### 3.1. Parametric Sweeps

A parametric sweep was carried out for the structural parameters illustrated in Figure 1, where *P* is the period, *t*_1_ is Au thickness, *t*_2_ is Si thickness, *A* is the width of the cross, *B* is the length of the cross, and *C* is the length of the Au patch. The goal was to determine the optimum parameters that can achieve the highest transmission (T) and a narrow FWHM, simultaneously at the operating wavelength of 4.28 μm. In these investigations, one parameter was swept while the other structural parameters were fixed. An x-polarized electric field was used as the input plane wave.

Figure 1b shows the transmission spectra as a function of wavelength for different periods *P*, while keeping other parameters constant at *A* = 0.3 µm, *B* = 2.2 µm, *C* = 2.2 µm, *t*_1_ = 0.1 µm, and *t*_2_ = 1.55 µm. A gradual redshift in the transmission spectrum is observed as *P* increases. Moreover, increasing *P* from 2.4 μm to 2.6 μm increases the transmission efficiency from 79% to 87%. The periodic arrangement of the hybrid metasurface induces coupling resonances between adjacent unit cells. Consequently, changing *P* affects the coupling of resonances among these unit cells, thereby influencing the FWHM [38]. As *P* increases, the spacing between the pillars enlarges, which reduces the coupling between neighboring pillars and results in a gradual widening of the FWHM [17,38]. Figure 1b demonstrates that increasing *P* from 2.4 μm to 2.6 μm leads to a broadening of the resonant peak FWHM from 40 nm to 46 nm. A period of *P* = 2.5 μm is selected as it optimally balances the highest transmission (87%) with the lowest FWHM (43 nm).

Figure 1c shows the transmission efficiency as a function of wavelength for different thicknesses *t*_1_. According to the principles of constructive interference and effective refractive index, changes in the hybrid metasurface thickness modify the optical path length, thereby shifting the spectral positions of the transmission peaks [41,42]. As demonstrated in Figure 1c, changes in *t*_1_ result in variations in the position of the transmission resonant. This demonstrates the tunability of the transmission response in the mid-infrared region (MIR) range by modifying *t*_1_, indicating the potential of this design for a variety of MIR spectroscopy applications. To further quantify this effect, a parametric sweep of *t*_1_ was performed while keeping the other parameters fixed at *P* = 2.5 μm, *A* = 0.3 μm, *B* = 2.2 μm, *C* = 2.2 μm, and *t*_2_ = 1.55 μm. The optimal thickness was found to be *t*_1_ = 0.1 μm, which yields the maximum transmission of 87% while maintaining the minimum FWHM of 43 nm.

Figure 1d presents the variation in transmission efficiency as a function of the thickness *t*_2_ at *P* = 2.5 µm, *A* = 0.3 µm, *B* = 2.2 µm, *C* = 2.2 µm, and *t*_1_ = 0.1 µm. The figure demonstrates that increasing *t*_2_ from 1.45 μm to 1.65 μm redshifts the transmission response, attributed to the increase in the effective optical path length. A thickness of *t*_2_ = 1.55 μm was selected, as it matches with the target wavelength of λ = 4.28 μm.

**Figure 2:**
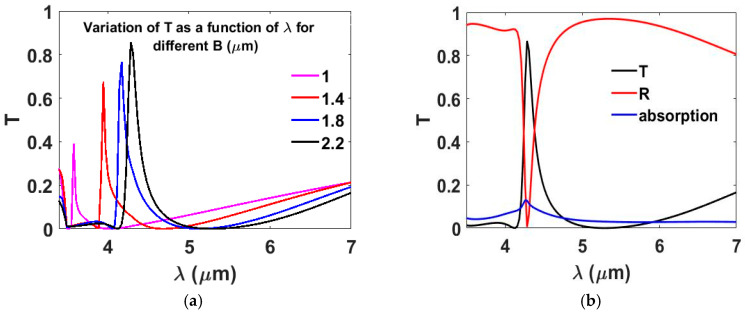
(**a**) Transmission *T* as a function of wavelength (λ) for various *B* values, highlighting the effect of geometry on spectral behavior. (**b**) Variation in R, T, and loss as a function of the λ showing that the proposed metasurface has low losses.

The effect of varying the cross-arm length *B* was further investigated, while keeping *P* = 2.5 µm, *A* = 0.3 µm, *C* = 2.2 µm, *t*_1_ = 0.1 µm and *t*_2_ = 1.55 µm. Figure 2a depicts the transmission variation versus wavelength for different *B* values. The results show that increasing *B* improves transmittance and shifts the optical response toward longer wavelengths, which can be attributed to the increased effective optical path length. Specifically, T increases from 39% at λ = 3.6 μm for *B* = 1 μm to 87% at λ = 4.28 μm for *B* = 2.2 μm. A cross-arm length of *B* = 2.2 μm was selected as it achieves the highest transmission efficiency. Further increasing *B* beyond 2.2 μm results in a slight red-shift and broadening of the resonance, which lowers spectral selectivity and does not yield a significant gain in transmission efficiency.

It is worth noting that varying the parameter *C* has only a minor influence on the transmission response within the studied range. However, when *C* is set equal to the period *P*, the gold patches merge into a continuous metallic film. In this scenario, the structure behaves as a reflective mirror, resulting in complete suppression of transmission and the disappearance of the designed resonance.

**Figure 3:**
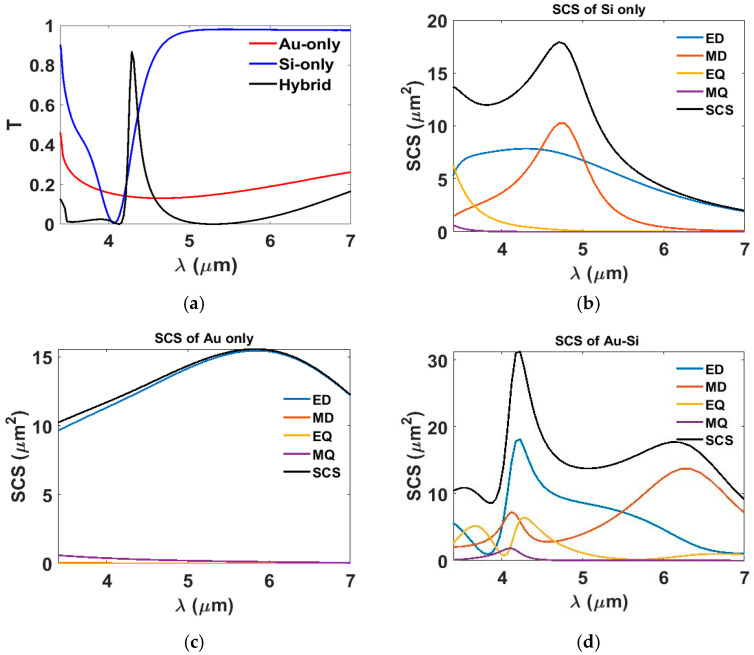
Depicts the influence of the hybrid metasurface on the transmission response. (**a**) *T* as a function of wavelength (λ) for Au-only, Si-only, and hybrid metasurfaces. The multipole expansion and the SCS of (**b**) the Si-only, (**c**) the Au-only, and (**d**) the hybrid metasurfaces.

Figure 2b illustrates the variation in reflection, transmission, and absorption (where absorption is calculated as 1 − R − T) as a function of λ at the optimized parameters: *P* = 2.5 µm, *t*_1_ = 0.1 µm, *t*_2_ = 1.55 µm, *A* = 0.3 µm, *B* = 2.2 µm, and *C* = 2.2 µm. Figure 3b shows that the incident light is largely reflected, with the exception occurring at the resonance wavelength λ = 4.28 µm, where minimal absorption is observed. Across the entire wavelength range, absorption remains below 20%, indicating minimal energy loss.

### 3.2. Physical Mechanism of the Resonance

To clarify the advantages of hybrid metasurfaces and the mechanism underlying their response, we analyzed the transmission of Au-only, Si-only, and hybrid structures by simulating the transmission response of each one at the optimum geometrical dimensions and calculating the SCS [43], as the transmission spectra are dictated by the SCS [44].

Figure 3a illustrates the transmission spectra for the Si-only, Au-only, and hybrid metasurfaces. Figure 3b illustrates that the Si-only metasurface exhibits strong transmission throughout much of the spectrum, except for a dip around λ = 4.28 μm. The corresponding SCS analysis indicates that this response is primarily governed by the ED and MD resonances, with a minor contribution from the electric quadrupole (EQ). In contrast, the Au-only metasurface exhibits a transmission of less than 20%, which is predominantly associated with the ED Mie resonance, as presented in Figure 3b.

As shown in Figure 3a, the hybrid metasurface achieves a high transmission efficiency of 87% at λ = 4.28 μm. The addition of the Si crosses to the Au patch array strongly modifies the transmission response, red-shifting the resonance and narrowing the bandwidth. This enhancement is attributed to the hybridization between the plasmonic resonances of the Au patches (characterized by confined surface currents) and the dielectric Mie resonances of the Si crosses. This coupling improves impedance matching with free space, suppresses reflection, and enhances forward scattering, resulting in the observed sharp and high-efficiency transmission peak. The coupling between the Au and Si components excites multiple Mie resonances, comprising ED, MD, EQ, and MQ modes, as depicted in Figure 3d.

**Figure 4:**
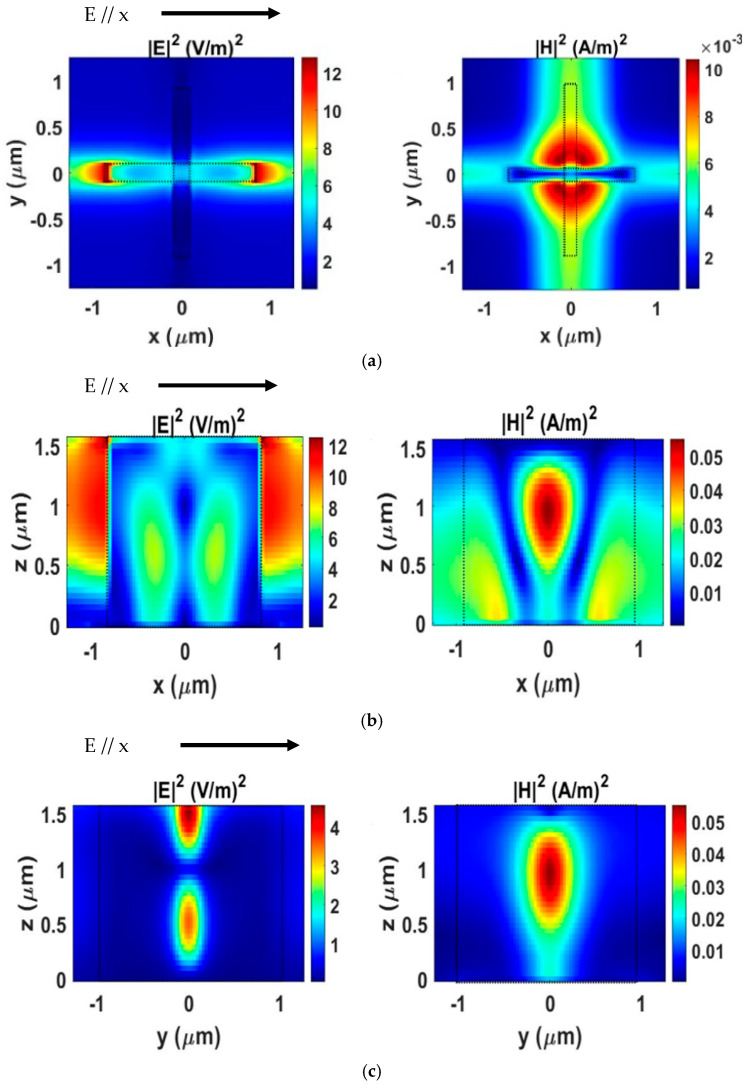
Electric (∣E∣^2^) and magnetic (∣H∣^2^) field profiles of the filter at the resonant wavelength (λ = 4.28 μm), evaluated for a single unit cell of the periodic structure shown in Figure 1a. (**a**) Field distributions in the x–y plane. (**b**) Field distributions in the x–z plane. (**c**) Field distributions in the y–z plane. The incident light is x-polarized, with the polarization direction (E // x) indicated by arrows in the plots. The results reveal strong localization of the E-field at the ends of the cross-arms and enhanced confinement of the H-field at the structure’s center.

Figure 4 shows the spatial distributions of the electric (|E|^2^) and magnetic (|H|^2^) field intensities at the resonant wavelength, evaluated at the Si–air interface indicated in Figure 1a. The field distributions are presented for the three orthogonal planes: *x*–*y*, *x*–*z*, and *y*–*z*. The left column corresponds to the electric fields, while the right column represents the magnetic fields. The magnetic field maps reveal a pronounced localization of the H-field, with a peak intensity in the middle of the structure. In parallel, the electric field distributions exhibit strong confinement at the termini of the cross-arms. This simultaneous increase in both electric and magnetic fields at the Si–air interface highlights the strong light–matter interaction within the metasurface. Such localized field intensities indicate that the filter exhibits strong sensitivity to variations in the refractive index of the surrounding medium, thereby offering significant potential for sensing applications. These field profiles also corroborate the SCS analysis in Figure 3b, confirming that the optical response is predominantly governed by the ED and MD resonances. Specifically, the strong E-field localization at the cross-arm ends reflects the ED contribution, while the enhanced H-field concentration at the structure center is characteristic of the MD resonance. This interpretation is consistent with multipole decomposition studies reported in the literature [45], where ED and MD modes are the dominant contributors in similar hybrid metasurfaces.

### 3.3. Influence of Incidence Angle on Transmission

**Figure 5:**
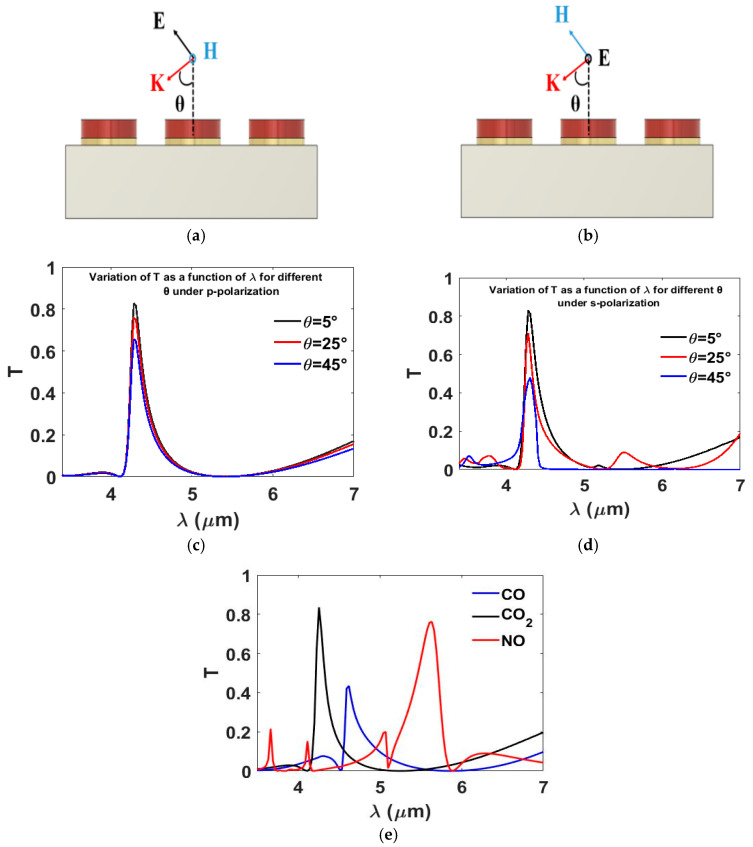
(**a**) Schematic of the proposed hybrid metasurface under p-polarization. (**b**) Schematic of the proposed hybrid metasurface under s-polarization. (**c**) Transmission spectra at different incidence angles (5°, 25°, 45°) under p-polarization. (**d**) Transmission spectra at different incidence angles under s-polarization. (**e**) Transmission spectra for various values of cross-arm width *A*, demonstrating spectral tunability of the filter across the MIR range.

Depending on intended application and optical system path, the incident light may not be necessarily normal to the filter. Hence, investigating the effect of incidence angle on the performance of the proposed hybrid metasurface is essential. Figure 5a,b shows the definition of the inclined incidence under p- and s-polarization, respectively. Figure 5c,d presents the transmission spectra as a function of λ for incident angles of 5°, 25°, and 45° under *p*- and *s*-polarizations, respectively. For *p*-polarized light, varying the incident angle between 5° and 45° impacts the transmission efficiency without affecting the spectral location of the resonance. The transmission efficiency is around 70% for an incident angle equal to 45°. For s-polarization, the incident angle affects the transmission efficiency only without any effect on the spectral position. However, increasing the incident angle to 45° produces a greater effect on transmission efficiency compared with *p*-polarization. Also, at an incident angle equal to 25°, there is an additional mode that is excited at λ ≈ 5.5 μm for the s-polarization. Overall, the proposed design has a high incident angle tolerance of up to 45° for the p-polarization and more than 25° for the s-polarization.

We further examined the tunability of the proposed filter design for gas detection applications by systematically adjusting its geometrical parameters. The results reveal that the transmission response of the hybrid filter can be accurately engineered to coincide with the mid-infrared (MIR) absorption bands of several environmentally and industrially relevant gases, including carbon dioxide CO_2_ at 4.2 µm, CO at 4.6 µm, and NO at 5.6 µm, as illustrated in Figure 5e. This spectral alignment is achieved through precise control of the cross-arm width (*A*), which was varied from 0.3 µm through 0.4 µm up to 1.15 µm. The demonstrated tunability underscores the versatility of the hybrid metasurface filter and highlights its strong potential for integration into highly selective MIR sensing platforms. Unlike conventional Fabry–Pérot or multilayer thin-film filters, which typically rely on complex multilayer stacks and offer limited tunability, the hybrid metasurface filter achieves spectral selectivity through simple geometrical modulation at the nanoscale. This unique design flexibility underscores the versatility of the proposed structure and highlights its strong potential for integration into highly selective and compact MIR sensing platforms.

To place the performance of our filter in context, Table 1 summarizes its specifications in comparison with representative Fabry–Pérot filters, guided-mode resonance devices, and plasmonic metasurfaces. The results show that the proposed hybrid design achieves a superior balance of high transmission, narrow bandwidth, and a competitive Q-factor, thereby outperforming previously reported approaches and highlighting its suitability for mid-infrared spectroscopy applications.

**Figure 6:**
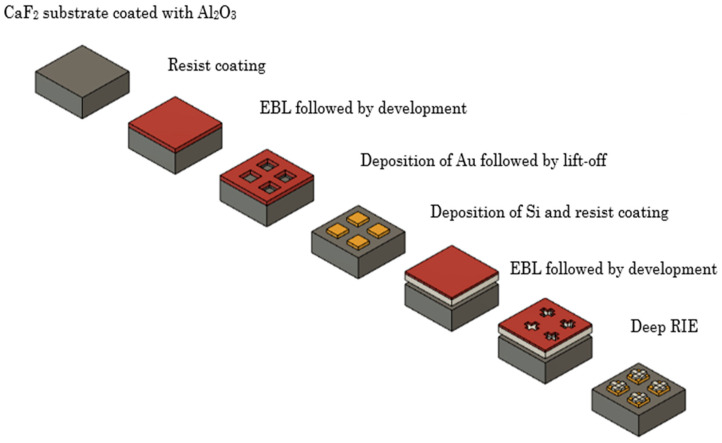
Schematic illustration of the fabrication process for the presented hybrid metal–dielectric metasurface.

Finally, a prospective fabrication scheme is presented to illustrate the feasibility of prototyping the hybrid metasurface design; this scheme represents a potential pathway and does not reflect an experimental realization in the present study. Figure 6 presents a schematic of a potential fabrication process for the proposed hybrid metasurface, which involves two electron-beam lithography (EBL) steps. As a first step, a 15 nm magnesium oxide (MgO) layer is deposited to serve as an adhesion layer for both Au and Si. MgO is selected due to its previously demonstrated effectiveness as an adhesion layer for various materials on CaF_2_ substrates. Importantly, incorporating the MgO layer into the simulated structure does not alter the transmission response of the device. Subsequently, the CaF_2_ substrate is spin-coated with an EBL resist. In this work, SML resist is recommended due to its proven suitability for achieving high-aspect-ratio patterns [47]. Au patches and alignment markers are then defined by EBL. The alignment markers play a critical role in ensuring accurate registration during the subsequent lithography step. Following exposure, Au is deposited using electron-beam evaporation, and lift-off is performed overnight to obtain the Au patches. This step is followed by the deposition of Si using plasma-enhanced chemical vapor deposition (PECVD), followed by coating the substrate again with an EBL resist. In the second EBL step, the Si crosses are defined using the previously defined alignment markers to guarantee precise overlay with the Au patches. After developing the resist, a 25 nm Al_2_O_3_ layer is deposited to function as the etch mask. Notably, the presence of this Al_2_O_3_ layer does not influence the simulated optical response. Finally, deep reactive ion etching (RIE) defines the Si cross structures.

It should be noted that the proposed process requires accurate alignment between the Au patches and the Si crosses. Modern EBL systems routinely achieve overlay accuracies within ±10–20 nm, which are sufficient for the sub-100 nm features of this design. Previous studies on hybrid metasurfaces have shown that misalignments of this magnitude typically result in only modest reductions in transmission efficiency and minor spectral shifts, without altering the fundamental resonance behavior.

## 4. Conclusions

In summary, a hybrid metal–dielectric metasurface tailored for mid-infrared (MIR) spectroscopy was numerically investigated. The proposed design exhibits both high transmission efficiency and a pronounced high-Q resonance, thereby offering strong spectral selectivity. The underlying physical mechanisms responsible for these transmission characteristics were elucidated through resonance and field distribution analyses. Moreover, the influence of key geometrical parameters on device performance was systematically examined, demonstrating the versatility and tunability of the structure. These results highlight the strong potential of the hybrid metasurface for MIR sensing and spectroscopy applications. Finally, a feasible fabrication strategy based on standard top-down nanofabrication techniques was proposed, underscoring the practical viability of implementing the design in experimental settings. The proposed metasurface architecture could be further developed for integration into compact on-chip photonic platforms, enabling real-time, highly selective gas detection and advancing the capabilities of MIR spectroscopy technologies.

## Figures and Tables

**Table 1:** Performance comparison of transmission-mode bandpass filters.

Filter Type	Transmission Efficiency	FWHM/Bandwidth	Q-Factor	Mode	Reference
This work (hybrid MD metasurface)	87%	43 nm	≈99.5	Transmission	—
Fabry–Pérot bandpass (GeSbTe phase-change)	60–70%	50–65 nm	70–90	Transmission	[6]
Guided-mode resonance (mid-IR)	~65–75%	~55 nm (4.25 µm)	~77	Transmission	[46]
Plasmonic metasurface bandpass (LWIR)	~40–50%	~1.5 µm	<10	Transmission	[10]

## Data Availability

Data will be made available on request.
